# Hemp biochar impacts on selected biological soil health indicators across different soil types and moisture cycles

**DOI:** 10.1371/journal.pone.0264620

**Published:** 2022-02-28

**Authors:** Idowu A. Atoloye, Ifeoluwa S. Adesina, Harmandeep Sharma, Kiran Subedi, Chyi-Lyi (Kathleen) Liang, Abolghasem Shahbazi, Arnab Bhowmik

**Affiliations:** 1 Department of Natural Resources and Environmental Design, North Carolina A&T State University, Greensboro, NC, United States of America; 2 Analytical Services Laboratory, College of Agriculture and Environmental Sciences, North Carolina A&T State University, Greensboro, NC, United States of America; 3 Center for Environmental Farming Systems, North Carolina A&T State University, Greensboro, NC, United States of America; De Montfort University Faculty of Health and Life Sciences, UNITED KINGDOM

## Abstract

Application of crop residues and biochar have been demonstrated to improve soil biological and chemical properties in agroecosystems. However, the integrated effect of organic amendments and hydrological cycles on soil health indicators are not well understood. In this study, we quantified the impact of hemp residue (HR), hemp biochar (HB), and hardwood biochar (HA) on five hydrolytic enzymes, soil microbial phospholipid (PLFA) community structure, pH, permanganate oxidizable carbon (POXC) soil organic carbon (SOC), and total nitrogen (TN). We compared two soil types, Piedmont and Coastal Plain soils of North Carolina, under (i) a 30-d moisture cycle maintained at 60% water-filled pore space (WFPS) (D-W1), followed by (ii) a 7-day alternate dry-wet cycle for 42 days (D-W2), or (iii) maintained at 60% WFPS for 42 days (D-W3) during an aerobic laboratory incubation. Results showed that HR and HB significantly increased the geometric mean enzyme activity by 1-2-fold in the Piedmont soil under the three moisture cycles and about 1.5-fold under D-W in the Coastal soil. In the presence of HA, the measured soil enzyme activities were significantly lower than control under the moisture cycles in both soil types. The shift in microbial community structure was distinct in the Coastal soil but not in the Piedmont soil. Under D-W2, HR and HB significantly increased POXC (600–700 mg POXC kg^-1^ soil) in the Coastal soil but not in the Piedmont soil while HA increased nitrate (8 mg kg^-1^) retention in the Coastal soil. The differences in amendment effect on pH SOC, TN, POXC, and nitrate were less distinct in the fine-textured Piedmont soil than the coarse-textured Coastal soil. Overall, the results indicate that, unlike HA, HR and HB will have beneficial effects on soil health and productivity, therefore potentially improving soil’s resilience to changing climate.

## Introduction

In the past five years, the cultivation of hemp [*Cannabis sativa* (L.)] for cannabidiol (CBD) has become a center of attention among farmers in the United States following the signing of the 2018 Farm Bill into law, which re-established it as an agricultural commodity when tetrahydrocannabinol (THC) concentration is below 0.3% [1,2]. This has led to an increase in the hectarage of cultivated CBD hemp from about 12,000 ha in 2018 to 59,000 ha in 2019 [3]. Hemp is a crop that accumulates biomass faster than other row crops and as such is efficient at capturing 15 tons of atmospheric carbon dioxide per hectare, which makes it a strong biosequestration candidate for building soil organic carbon (SOC) [2]. As an emerging crop among farmers, the high biomass from CBD hemp can be leveraged, via conversion to biochar, to enhance soil health and nutrient cycling in the face of increasing climate variability as the return of crop residues has been deemed desirable to mitigate SOC loss and greenhouse gas emission [4].

With the disruption in agricultural practices due to climate change, maintaining soil health and nutrient cycling is critical for sustainable agriculture. Soil water is one of the salient environmental factors that regulate both biotic and abiotic processes in soils [5,6]. Repeated dry/wet (D-W) cycles and longer periods of moisture inundation are predicted to occur more frequently because of global climate change [7,8]. Multiple D-W cycles have been found to result in either reduced decomposition of SOC and microbial activities [9] or mobilization and metabolism of otherwise inaccessible soil carbon (C) by increasing soluble C release, microbial biomass growth, and activity [8,9]. Pezzolla et al. [10] reported during a 14-d experiment, nitrogen (N) mineralization and water-extractable phosphorus (P) in unamended soils were higher under repeated D-W cycles than constant moisture. Other studies reported no difference between the effects of repeated D-W cycles and constant moisture on N mineralization indicating that D-W cycles do not always cause shriveling of microbial cells [6,11]. Elsewhere, a study by Sibylic et al. [12] showed that the direct impact of soil moisture stress decreased the activity of P cycling enzymes than C cycling, while Li et al. [13] reported that the activity of C cycling enzymes was greatly reduced than N cycling enzymes in inorganically fertilized soils. Thus, a better understanding of moisture cycles on soil biological and chemical processes is needed for improved management decisions, especially in managed agricultural systems.

Research on the interactive effects of biochar and D-W cycle on soil microbial functional activity and SOC dynamics are sparse. Biochar properties are unique depending on the feedstock, and pyrolysis temperature, and type [14]. As such, changes in SOC decomposition and microbial functioning following biochar application under different moisture cycles would depend greatly on biochar characteristics. A 63-d incubation study by Song et al. [15] observed little or no differences in C mineralization between soils undergoing repeated D-W cycles and under constant moisture following the application of wheat straw biochar produced at 300°C. However, they observed significantly lower C mineralization in soils under D-W cycles compared to constant moisture when wheat straw biochar produced at 600°C was applied. In another study, Rahman et al. [9] found that the application of maize straw biochar produced at 400°C did not mitigate the declining effect of D-W cycles on soil respiration because biochar did not affect the soil aggregation. Therefore, there is still a need to look at how the interaction between biochar and D-W would impact soil biological and biochemical properties. In addition, the interactive effects of amendments and D-W cycles on soil microbial nutrient cycling would also be regulated by soil properties such as e.g., pH, texture [16].

Differences in soil texture contribute significantly to the variability of amendments on soil biochemical processes [11]. Soils with higher sand fractions are thought to experience more disruption in nutrient cycling processes due to lower water potential and rapid aggregate disruption unlike soils with higher clay content [17]. For instance, Harrison-Kirk et al. [16] found that silt loams soils were more susceptible to N losses compared to clay loam soil when exposed to D-W cycles. However, another study reported that N mineralization was greater in silt loam soil followed by loam and then sandy loam soil [11]. Besides, for a specific soil type, the use of amendments of varying qualities can differentially shape the response of soil microbes to D-W cycles due to regulatory effects on soil physical (e.g., aggregate formation, soil water retention, substrate diffusion) and chemical properties (e.g., cation exchange capacity, nutrient availability) [11,16]. As a result of differences in the interactive effects of D-W cycles and amendments in different soil types, the response of microbial community structure and function remains uncertain. Under global climate change, it becomes imperative to further understand the interactive effects of amendments and D-W cycles on the composition and biochemical functions of soil microbes in different soil types with the view to avoiding negative effects on soil biogeochemical nutrient cycling and crop yield.

Therefore, for a crop (hemp) known for its high biomass production, it is important to examine the effects of hemp residue and hemp-derived biochar amendments on soil health indicators under different D-W events in soils with contrasting properties. In this study, we examined how the application of hemp residue, hemp, and hardwood biochar would affect soil microbial community structure, enzymatic activity, and multiple chemical and biochemical [pH, permanganate oxidizable carbon (POXC), nitrate-N, total SOC, total nitrogen (TN)] soil health indicators in response to hydrological cycles in two soil types located in North Carolina, USA.

## Materials and methods

### Soil collection and analyses

Soil (0–15 cm) for this study was collected in March 2019 from the North Carolina Agricultural and Technical (N.C. A&T) State University Research Farm in Greensboro (36°5′N, 79°44′W) and the Small Farm Unit of the Center for Environmental Farming Systems (CEFS) in Goldsboro (35°23′N, 78°5′W), North Carolina. The soil from N.C. A&T, which is in the Piedmont region and hereinafter mentioned as “Piedmont”, was previously cultivated under hemp in 2018 summer before rye (*Secale cereale*) and crimson clover (*Trifolium incarnatum*) as winter cover crops, while the soil collected at CEFS, which is from the Coastal Plain and hereinafter mentioned as “Coastal”, was previously cultivated under buckwheat (*Fagopyrum esculentum*) in summer followed by rye and crimson clover as winter cover crops. Piedmont soil is a sandy clay loam soil classified as fine, mixed, active, thermic Ultic Hapludalfs Alfisol, while Coastal soil is loamy sand soil classified as fine-loamy, siliceous, subactive, thermic Aquic Paleudults Ultisols. The 60 yr average annual precipitation and temperature are 1291 mm and 16°C, respectively for the Coastal soil and 1179 mm and 15°C, respectively for the Piedmont soil.

Soil samples were passed through a 2 mm mesh sieve, visible crop residues were removed, and sieved soils were stored at 4 ˚C for 7 days prior to incubation set up. A portion of the soil was air-dried to determine the pH [(soil:water (1:5, w/v]), total SOC, TN, and Mehlich (III) P. Nitrate-N (NO_3_^-^-N) was determined using field moist samples. The soil properties are presented in Table 1.

**Table 1:** Initial soil physical and chemical properties.

	Coastal	Piedmont
Physical		
Soil texture, g kg^-1^	Loamy sand	Sandy clay loam
Sand	820^†^	580
Silt	80	130
Clay	100	290
Chemical		
pH	5.6 (0.02)	7.2 (0.03)
Total organic carbon, g kg^-1^	17.8 (0.05)	14.8 (0.14)
Total nitrogen, g kg^-1^	1.9 (0.02)	2.1 (0.04)
Mehlich 3 Phosphorus mg kg^-1^	338 (5.24)	182 (1.39)
Nitrate nitrogen (mg kg^-1^)	24.3 (0.21)	54.5 (1.36)

^†^Value obtained from USDA-NRCS soil survey data.

### Soil amendments preparation and characterization

Hemp residues from the 2018 harvest season were collected from N.C. A&T research farm in Greensboro. Hardwood biomass was obtained from an oak tree (*Quercus* sp.). The hemp and hardwood biochar were produced using slow pyrolysis at a temperature of 800 ˚C for 1 h. Elemental analysis of biochar samples for C, H, and N was determined using a CHN analyzer 2400 series II, (Perkin/2400 Elmer, Akron, OH, USA), while the chemical analysis was determined after digestion with HNO_3_/HF using an Optima 8300 Inductively coupled plasma—optical emission spectrometry (Perkin-Elmer, Inc. Shelton, CT, USA). The Brunauer-Emmett-Teller (BET) surface area analysis of the biochar was carried out using the ASAP 2020 BET Surface Area and Porosity Analyzer (Micromeritics, Norcross, GA, USA) at the Joint School of Nanoscience and Nanoengineering at N.C. A&T State University. The chemical properties of the amendments are presented in Table 2. The amendments vary in their C:N ratio (C_total_:N_total_), i.e., 46.7, 67.6, and 229 for hemp residue (HR), hemp biochar (HB), and hardwood biochar (HA), respectively. The BET surface area of the hemp biochar is 73 m^2^ g^-1^ while the hardwood biochar has a value of 656 m² g^-1^.

**Table 2:** Total elemental analysis of the amendments.

Property	Hemp residue	Hemp biochar	Hardwood biochar
pH (1:10, soil/water)	6.3	9.9	9.9
EC dS/m	2.83	1.38	0.77
C (%)	41.6	70.7	82.6
H (%)	5.77	2.32	1.19
N (%)	1.04	1.22	0.42
C_total_:N_total_	46.7	67.6	229
H:C	1.66	0.39	0.17
Phosphorus (%)	0.43	0.59	0.09
Potassium (%)	0.89	1.44	0.14
Calcium (%)	0.92	1.99	0.9
Magnesium (%)	0.26	0.35	0.57
Iron (mg kg^-1^)	93.6	655	2656

### Incubation experiment

**Fig 1:**
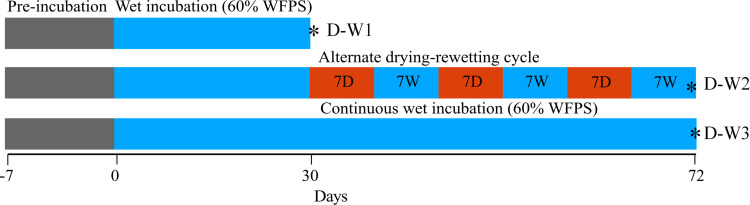
Schematic representation of the experimental setup. Blue squares represent the wet incubation at 60% WFPS and red-shaded blocks represent the drying period. The samples were collected at the end of days 30 and 72. D, drying phase; W, rewetting phase; WFPS, water-filled pore spaces. D-W1, 30 d 60% WFPS; D-W2, 30 d 60% WFPS followed by 7 d alternate D-W for 42 d; D-W3, 72 d 60% WFPS. * Indicates sampling points.

Four treatments were used for the incubation study. This includes soils amended with (i) HR, (ii) HB, (iii) HA, and (iv) control (no amendment). A soil microcosm laboratory incubation experiment was designed under varying moisture cycles, i.e., (i) a 30-d moisture cycle maintained at 60% water-filled pore space (WFPS) (D-W1); followed by (ii) a longer moisture cycle of a 7-d alternate dry-wet (5 and 60% WFPS) cycle for 42-d (D-W2); or (iii) a 42-d moisture cycle maintained at 60% WFPS (D-W3) (Fig 1). The experiment was replicated three times making a total of 72 samples. Before the addition of the amendments, 50 g of soil (dry weight basis) were preincubated at 40% WFPS in 100 mL specimen cups for one week at 25 ˚C [18]. Following the preincubation period, five grams, equivalent to 10% rate w/w, of each amendment were added to the soil samples and re-adjusted to 60% WFPS using deionized water. The samples were thoroughly mixed to ensure the even distribution of moisture and the amendments after which they were repacked to maintain a bulk density of 1.2 g cm^-3^. All jars were randomly placed in an incubator at 25°C. For the 7-day alternate drying and rewetting, the soils were gently rewetted to minimize disturbance following a drying period (25°C). The soils were destructively sampled at the end of each moisture cycle for further analysis as mentioned below.

### Potential enzyme activities and soil microbial community structure

The potential activities of five soil hydrolytic enzymes, β-glucosidase, β-glucosaminidase, acid phosphatase, phosphodiesterase, and arylsulfatase, associated with C, N, P, and S cycling, respectively, were determined [19–22]. Briefly, 1 g of air-dried soil was incubated at 37 ˚C with 4 mL of the appropriate buffer and 1 mL of substrate for each enzyme for 1 h (Table 3). The assay included a control to which the substrate was added after incubation. Following the incubation, the reaction was stopped with 1 mL of 1.0 M CaCl_2_ and 4 mL of 0.5 M NaOH or 0.1 M Tris(hydroxymethyl)aminomethane buffer depending on the enzyme, and the suspension was filtered using a Whatman No. 2v filter. The *p*-nitrophenol product released was measured colorimetrically at 400 nm using a visible spectrophotometer. Soil microbial community structure, using phospholipid fatty acids (PFLA) analysis, was determined using fresh soil at the Ward Laboratories Inc. (Kearney, NB). Briefly, soil lipid was extracted with one-phase chloroform:methanol:phosphate (1:2:0.8) buffer, with PLFA eluted from silicic acid columns in methanol, following removal of non-polar lipids by washing with chloroform and acetone [23]. An aliquot of the total PLFA extract was used to estimate viable biomass by colorimetric assay for phospholipid phosphate [24]. Microbial community structure was described by the fatty acid composition of the PLFA in the soil. Fatty acids were summed up into groups of biomarkers according to [25], which are: Gram-negative [G (-)]bacteria, cy17:0 and cy19:0; Gram-positive [G (+)] bacteria, iso and anteiso saturated branched fatty acids (i15:0, a15:0, i16:0, a16:0, i17:0, and a17:0); fungi, 18:2 ω6 cis and ratio of saturated to unsaturated PLFA. Microbial biomass was quantified by summing up all the identified PLFAs. The total bacterial biomass was represented by the sum of the biomarker PLFAs for both G (+) and G (-) bacteria.

**Table 3:** Reagents used for extracellular enzymes assay.

Enzyme	Substrate	Buffer	Stop solutions
β-glucosidase (E.C. 3.2.1)	0.05 M *p*-Nitrophenyl-β-d-glucopyranoside; CAS (2492-87-7)	MUB pH 6.0; 4 mL	0.5 M CaCl_2_; 0.1 M THAM (pH 12)
β-glucosaminidase (E.C. 3.2.1.30)	0.01 M *p*-Nitrophenyl-N-acetyl-β-D-glucosaminide; CAS (3459-18-5)	0.1 M acetate pH 5.5; 4 mL	0.5 M CaCl_2_; 0.5 M NaOH
Acid phosphatase (E.C.3.1.3.2)	0.05 M *p*-Nitrophenyl phosphate; CAS (333338-18-4)	MUB pH 6.5; 4 mL	0.5 M CaCl_2_; 0.5 M NaOH
Arylsulfatase (E.C. 3.1.6.1)	0.05 M *p*-Nitrophenyl sulfate; CAS (6217-68-1)	0.5 M acetate pH 5.8; 4 mL	0.5 M CaCl_2_; 0.5 M NaOH
Phosphodiesterase (E.C. 211-434-7)	0.005 M Bis-*p*-nitrophenyl phosphate; CAS (4043-96-3)	0.05 M THAM pH 8.0; 4 mL	0.1 THAM-0.5 M; NaOH pH 12

MUB, modified universal buffer; THAM, tris-hydroxymethl-aminomethane.

### Ancillary chemical and biochemical soil health indicators

Soil pH, a chemical soil health indicator, (1:5, soil:water w/v) was measured with a benchtop Thermo Orion 420A Plus pH meter (Thermo Scientific, Waltham, MA, USA). Inorganic nitrate-nitrogen (NO_3_^-^-N), a chemical soil health indicator, was determined in 2 M KCl extracts (1:5, w/v) after shaking at 180 rev min^-1^ for 1 h and filtered using Q2 filter papers (Fisher Scientific, Pittsburg, PA, USA). The NO_3_^-^-N concentration was determined colorimetrically using the Vanadium (III) acid method at 540 nm [26] using a Synergy HTX multimode microplate reader (Biotek Instruments Inc., Winooski, VT, USA). The POXC, a biochemical soil health indicator, was measured as described by [27]. Briefly, 20 mL 0.02 mol L^-1^ KMnO_4_ solution was added to 2.5 g of air-dried soil and placed on a shaker for 180 rev min^-1^ for 2 min. Thereafter, the soil was left undisturbed for 10 min and 0.5 mL of the supernatant was quantitively transferred into a second 50 mL centrifuge tube containing 49.5 mL of deionized water. The absorbance values were read at 550 nm using a Synergy HTX multimode microplate reader. The TC and TN, which are soil biochemical indicators, were measured using a CHN analyzer 2400 series II, (Perkin/2400 Elmer, Akron, OH, USA). The TC is equivalent to the total SOC because these soils did not contain carbonates.

### Statistical analysis

Statistical analysis was completed using a three-way analysis of variance (ANOVA) as a completely randomized design with the GLIMMIX procedure in SAS Studio University Edition (version 9.4, SAS Institute, Cary, NC, USA). The fixed factors were soil type, moisture cycle, and amendment type. The data was assessed to meet the assumptions of normality and constant variance, log-transformed if required and back-transformed. Differences between means were separated using Tukey-Kramer method (p<0.05). Where the three-way interaction (*F*-statistic) was not significant, a simple effect comparison of the interaction (LSMESTIMATE) was done. Pearson correlation test was performed to test the linear relationships (p<0.05) among the response variables for each soil type using the CORR procedure in SAS Studio University Edition. To distinguish the treatment effects on the response variables as a function of the soil type, amendment, and moisture cycle, principal component analysis (PCA) was performed after standardizing the data using the JMP package of SAS 9.3 (SAS Institute, Cary, NC, USA).

## Results and discussion

### Soil enzyme activities: Carbon, nitrogen, phosphorus, and sulfur enzymes

**Fig 2:**
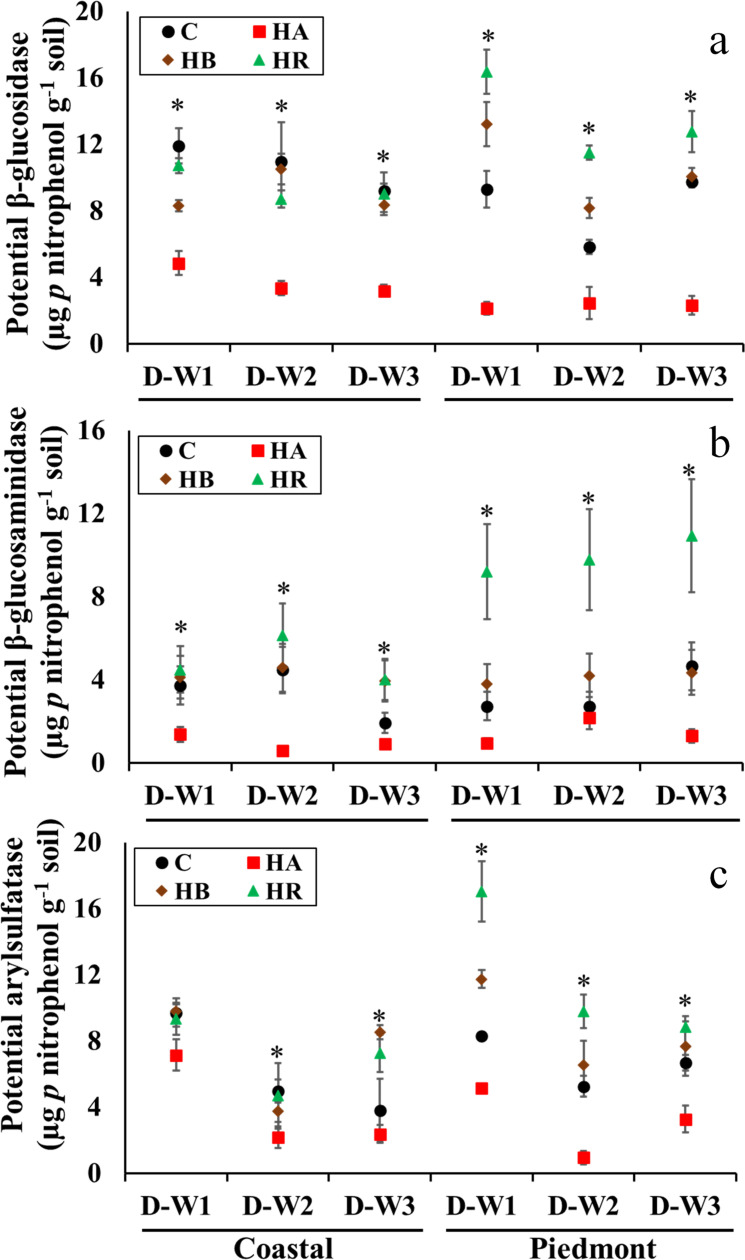
The interaction of soil type × moisture cycle × amendment for the potential β-glucosidase, β = glucosaminidase and acid phosphatase (panels a, b and c, respectively) in the amended soil mesocosms. Asterisk indicates difference among treatments (α = 0.05), based on three-way ANOVA. Error bars represent standard errors (n = 3). C, control; HR, hemp; HB, hemp biochar; HA, hardwood biochar; D-W1, 30 d 60% WFPS; D-W2, 30 d 60% WFPS followed by 7 d alternate D-W for 42 d; D-W3, 72 d 60% WFPS.

**Fig 3:**
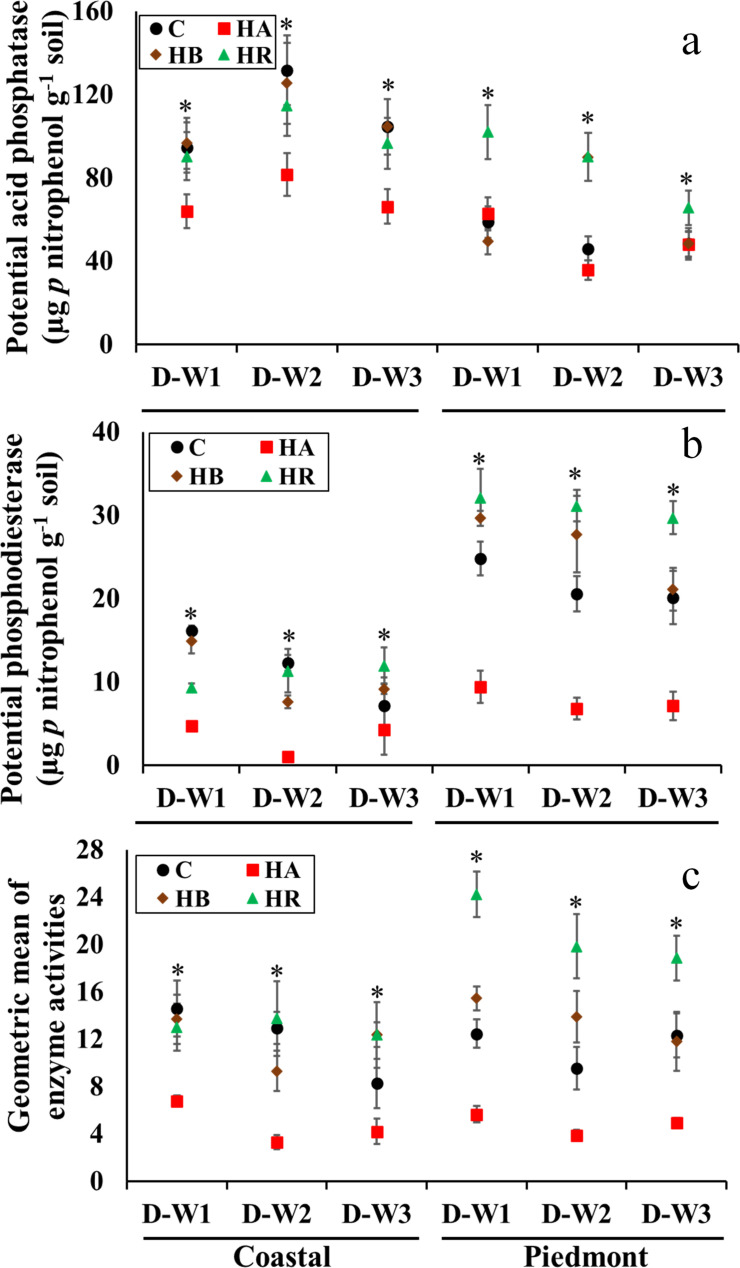
The interaction of soil type × moisture cycle × amendment for the potential arylsulfatase, phosphodiesterase, and geometric mean of enzyme activities (panels a, b and c, respectively) in the amended soil mesocosms. Asterisk indicates difference among treatments (α = 0.05), based on three-way ANOVA. Error bars represent standard errors (n = 3). C, control; HR, hemp; HB, hemp biochar; HA, hardwood biochar; D-W1, 30 d 60% WFPS; D-W2, 30 d 60% WFPS followed by 7 d alternate D-W for 42 d; D-W3, 72 d 60% WFPS.

Soil enzymes have been demonstrated to be good indicators of soil health [28,29]. The activities of measured soil enzymes were different as indicated by a significant amendment × soil type × moisture cycle interaction (Figs 2 and 3). Overall, the application of HA consistently reduced soil enzyme activities, while HR and HB had varying effects depending on soil type and moisture cycle. Thus, the effect of biochar on soil microbial functions is reliant on soil type, biochar type, and prevailing soil moisture [30]. The beneficial effect of either HR or HB on C cycling was reflected in the Piedmont soil to a greater extent than in the Coastal soil (Fig 2A). In the Coastal soil, β-glucosidase activity was not significantly different between the control (11.9 μg *p*-nitrophenol g^-1^ soil) and HR-amended soil (10.7 μg *p*-nitrophenol g^-1^ soil) but higher than in HB-amended soil (8.3 μg *p*-nitrophenol g^-1^ soil) at D-W1 (Fig 2A). However, by the end of D-W2 and D-W3, the β-glucosidase activity was similar among control, HR- and HB-amended soils (8.83 and 9.45 μg *p*-nitrophenol g^-1^ soil). Similarly, in the Piedmont soil, HA significantly reduced the β-glucosidase activity (2.32 μg *p*-nitrophenol g^-1^ soil), while it was 75–97% higher in HR-amended soil and about 40% higher in HB-amended soil compared to the control at D-W1 and D-W2. At D-W3, the β-glucosidase activity in HR-amended soil (12.1 μg *p*-nitrophenol g^-1^ soil) was higher than in control (9.76 μg *p*-nitrophenol g^-1^ soil) and HB-amended (10.1 μg *p*-nitrophenol g^-1^ soil) Piedmont soil (Fig 2A).

Furthermore, the effect of HR and HB on β-glucosaminidase, arylsulfatase, acid phosphatase, and phosphodiesterase varied with soil type and moisture cycle. In the Coastal soil, there was less distinction between HR and HB on the soil enzyme activities except for β-glucosaminidase at D-W3 (Fig 2B) and phosphodiesterase at D-W1 (Fig 3B). For the Piedmont soil, the effect of HR on the enzyme activities was consistently higher than HB except at D-W3 for arylsulfatase and acid phosphatase and D-W1 and D-W2 for phosphodiesterase. As such, the interactive effects of biochar and moisture cycle on soil enzymes varied more in the clayey soil. Bonette et al. [30] showed higher phosphatase activity in clay loam soil compared to sandy loam soil following the application of Miscanthus biochar during D-W cycles. Similar results were found by Copper and Warman [31] who showed that amendments increased C cycling enzymes in clayey soil than sandy soil. A plausible reason could be due to better formation of aggregates in the clay Piedmont soil unlike in the sandy Coastal soil which can result in increased stabilization of C substrates and efficient C cycling [32–34]. Also, soil enzymes activities have been strongly linked to soil characteristics such as pH [16,31,35,36]. The circumneutral pH and clay texture of the Piedmont soil might have favored higher microbial C cycling [16]. Furthermore, soils with higher clay content have been associated with increased enzyme activity due to stable clay-organic matter complexes, higher water retention ability, as well as increased protection of organic matter in soil aggregates [37]. Hence, the adaptive ability of soil microorganisms to moisture stress in the presence of amendments is different and likely to be lower in sandy soils compared to soils with higher clay content due to reduced nutrient cycling.

Considering the geometric mean of enzyme activities, we estimate that the application of HA reduced the overall enzyme activities in the Coastal soil by 40–80% and in the Piedmont soil by 45–60% (Fig 3C). The rate of biochar used in our study is higher compared to other studies [7,9] but similar to the rate used by Bonette et al. [30]. Consequently, the varying effect of HB and HA on soil enzyme activities shows that it is possible to increase both C sequestration and soil microbial enzyme activities without compromising one for another. Similar results were reported by Bonnett et al. [30] showing that application of dried cattle manure biochar at 10% w/w consistently reduced phosphatase activity in sandy loam and clay loam soil whereas the Miscanthus biochar applied at the same rate increased phosphatase activity, even though, the dried cattle manure biochar in the study by Bonnet et al. [30] had a lower C:N ratio (18) compared to Miscanthus biochar (84).

The contrasting effect of biochar on soil enzyme activities decomposition may have been driven by differences in (i) the change in soil physical conditions, (ii) the amount of readily decomposable C, or (iii) the presence of toxic compounds such as polycyclic aromatic hydrocarbons, polar pyrolysis condensates or residual pyrolysis oil [16,38,39]. Higher concentrations of toxic compounds create an unfavorable environment for microbes which can result in the disruption of microbial physiological functions, cellular membranes, denaturation of proteins, and SOM decomposition [40]. Additionally, the higher amount of C relative to N and P in HA is another factor that can cause a limitation in microbial utilization of C substrates, which was not observed by [30]. In the absence of N and P, microbial utilization of C is known to be limited due to an imbalance in nutrient stoichiometry.

While the effects of HR and HB on overall enzyme activities were less distinct in the Coastal soil except under D-W3, the geometric mean of enzyme activities in the Piedmont soil was consistently highest in HR-amended soils followed by HB-amended soils, except at D-W3 where there was no significant difference between HB-amended and control soils (Fig 3C). Microbially favorable substrates are likely dominant in HR- and HB-amended soils and this affected the responses of microorganisms to moisture stress. We were not able to find studies evaluating differences in crop residue and its biochar under different moisture cycles on soil enzyme activity. Rahman et al. [9] studied the effect of maize straw and maize straw biochar in a 56-d laboratory incubation and found that maize straw enhanced soil respiration under D-W cycles while maize straw biochar had no effect in clay loam soil. Our results showed that differences in effects of HR and HB were not distinct in the loamy sand, however, such was not the case in the sandy clay loam. As such, whether straw residue or its biochar would mitigate the effects of D-W cycles would depend on the soil type. An increase in enzyme activity under D-W conditions is beneficial for agroecosystem productivity.

### Variation in soil microbial community PLFAs concentrations

**Fig 4:**
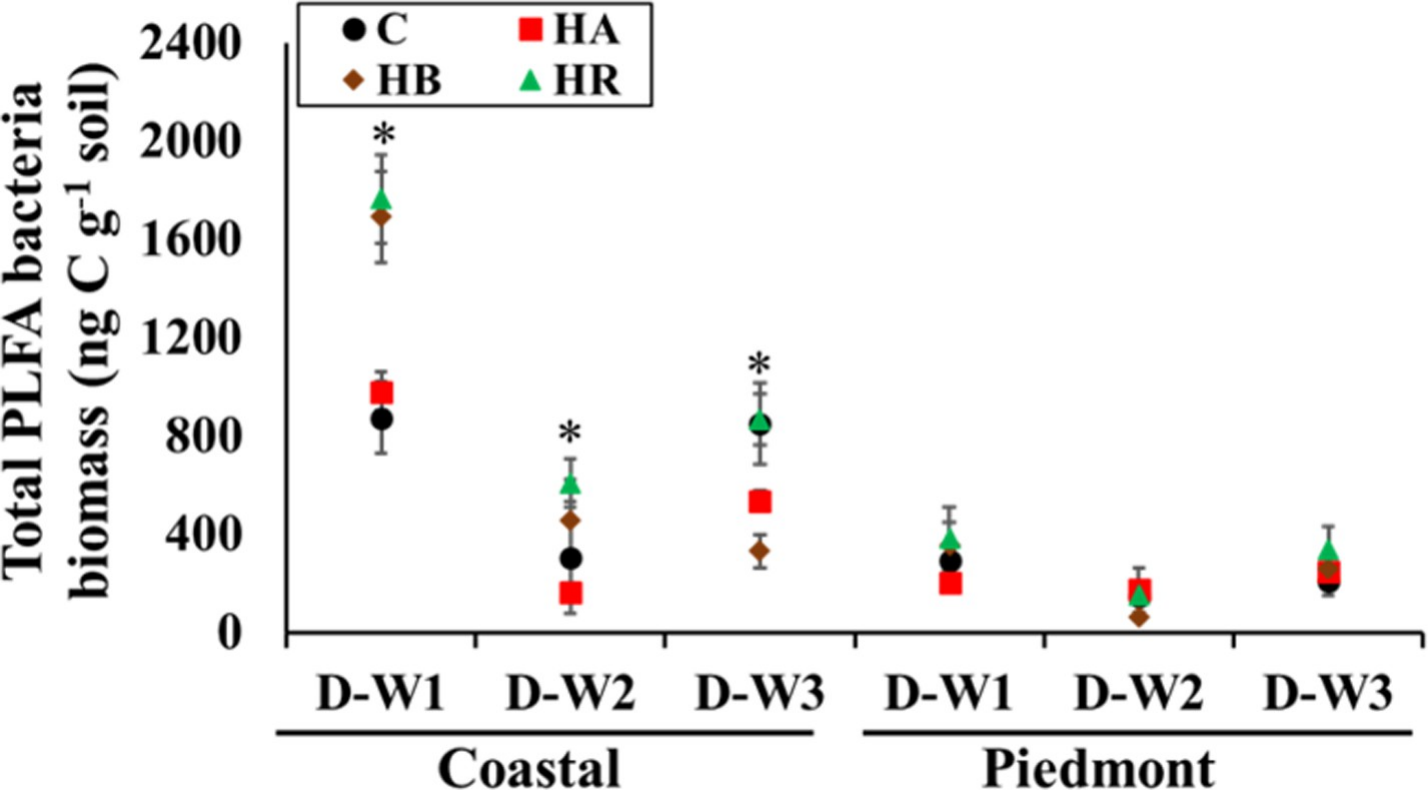
The interaction of soil type × moisture cycle × amendment for the total PFLA bacteria biomass in the amended soil mesocosms. Asterisk indicates difference among treatments (α = 0.05), based on three-way ANOVA. Error bars represent standard errors (n = 3). C, control; HR, hemp; HB, hemp biochar; HA, hardwood biochar; D-W1, 30 d 60% WFPS; D-W2, 30 d 60% WFPS followed by 7 d alternate D-W for 42 d; D-W3, 72 d 60% WFPS.

The PLFA microbial concentrations responded to a different degree than did soil enzymes across the soil types. The two-way interactions of amendment × moisture cycle (p = 0.011) and soil type × moisture cycle (p = 0.004) were significant for the total PLFA microbial biomass. Changes in the concentrations of total microbial, bacterial, and fungal PLFAs biomass were more distinct in the Coastal soil as compared to the Piedmont soil (Table 4 and Fig 4). In the Coastal soil, the concentration of total PLFA biomass was lowest in HA-amended soils across all moisture levels while there were no statistical differences (p>0.05) among treatments in the Piedmont soil. This indicated that active microbial communities between the soils are likely to be different (Table 4), thus corroborating the observed differences in enzyme activities.

**Table 4:** Means (*n* = 3) for total PLFA biomass, total PLFA fungi biomass, fungi:bacteria ratio, G (+): G(-), and sat:unsat for interaction effects (soil type × day × amendment) in soil mesocosms. Lower case letters designate LSMeans comparisons. All statistics are presented when treatment effects were significant (α = 0.05).

Effect		Total PLFA biomass	Total PFLA fungal biomass	F:B	G (+): Gram (-)	Sat:Unsat
		ng C g^-1^ soil				
Soil type (S)	Amendment (A)					
Coastal	C^†^	2209a	29.2b	0.049b	4.24a	6.86a
	HA	1443b	37.0ab	0.079a	2.85b	6.04ab
	HB	1758a	48.7ab	0.081a	2.32b	5.04ab
	HR	2622a	59.2a	0.062b	2.04b	4.37b
Piedmont	C	757	11.2	0.052	2.33	8.53
	HA	649	14.5	0.067	2.54	7.46
	HB	630	11.6	0.059	1.90	7.81
	HR	780	12.7	0.047	2.40	7.44
Moisture cycle (D)	Amendment					
D-W1	C	1214b	13.2	0.027	4.81	6.81
	HA	1319ab	14.8	0.034	3.76	7.04
	HB	1938a	23.2	0.031	3.30	6.30
	HR	1966a	27.4	0.035	3.18	5.60
D-W2	C	1422a	12.8	0.053ab	2.50	10.8
	HA	625b	20.0	0.099a	2.56	8.52
	HB	810ab	12.6	0.064a	1.73	11.1
	HR	1057a	9.2	0.028b	1.95	8.98
D-W3	C	1254a	34.9b	0.088b	2.58	6.08a
	HA	1100ab	41.8b	0.117ab	2.02	5.03ab
	HB	743b	46.2ab	0.164a	1.62	3.51b
	HR	1408a	81.6a	0.156a	1.74	3.68b
Soil type	Moisture cycle					
Coastal	D-W1	3328a	36.7	0.029b	3.77a	4.77b
	D-W2	1381b	39.2	0.113a	2.40b	6.73a
	D-W3	1633b	51.7	0.088a	2.30b	5.16ab
Piedmont	D-W1	742	9.6b	0.034b	3.66a	8.62b
	D-W2	631	4.4c	0.028b	1.94b	14.3a
	D-W3	736	45.4a	0.186a	1.67b	3.86c
ANOVA *p*-values						
A		**0.019**	0.434	0.103	**0.019**	0.151
D		**0.000**	**< .0001**	**< .0001**	**< .0001**	**< .0001**
S		**< .0001**	**< .0001**	0.175	0.059	**0.000**
S*A		0.491	0.533	0.724	0.062	0.506
D*A		**0.011**	0.345	**0.046**	0.979	0.425
S*D		**0.004**	**0.000**	**< .0001**	0.470	**< .0001**
S*A*D		0.245	0.487	0.555	0.424	0.594

^†^C, control; HR, hemp residue; HB, hemp biochar, HA, hardwood biochar; D-W1, 30 d 60% WFPS; D-W2, 30 d 60% WFPS followed by 7 d alternate D-W for 42 d; D-W3, 72 d 60% WFPS; PLFA, phospholipid-derived fatty acids; F:B, fungi biomass to bacteria biomass ratio; G+:G- BIOM, ratio of gram-positive to gram-negative bacteria biomass; sat:unsat, ratio of saturated to unsaturated fatty acids.

Long-term D-W events and constant moisture reduced the concentration of total and bacterial PLFA biomass by approximately 50–60% in the Coastal soil but not in the Piedmont soil (Table 4). In D-W1, both HR and HB increased the concentrations of total bacterial PLFA in the Coastal soil but by the end of D-W2, the total bacterial biomass in HB-amended soil was no longer different from the control and was lowest by the end of D-W3 (Fig 4). Essentially, amendment- and moisture-induced stress conditions did not produce significant shifts in bacterial communities in the clay Piedmont soil, indicating the likelihood of fewer disruptions in nutrient cycling, which is beneficial to agroecosystem productivity. As observed for bacterial PLFAs, the concentrations of the fungal PLFAs in the Coastal soil were highest in HR-amended soils but were not different from HB- and HA-amended soils except from control soils (Table 4). Averaged across soil types, the concentrations of fungal PLFAs among treatments were only different at D-W3, with the highest concentration present in HR-amended soils followed by HB-amended soils. While there were no statistical differences (p>0.05) among moisture events in the Coastal soil, the fungal PLFA concentration was highest in D-W3 and lowest at D-W2 in the Piedmont soil, indicating that stress due to drying-rewetting cycles negatively affected fungal communities in soils with higher clay content irrespective of amendment use (Table 4).

Soils with lower fertility are likely to experience a higher frequency of fluctuations in the active soil microbial biomass due to lower labile C and/or available nutrients when perturbed. Our results are in line with those of [41], suggesting that biochar amendment led to higher microbial biomass response in sandy soil than in clay soil. Zhang et al. [42] also found that bacterial communities in soils with lower pH values (<7) experienced significant changes but not in alkaline soils pH (>7) following the application of biochar produced from peanut shells. Microbial composition and sensitivity to perturbations are known to be different depending on soil chemical and physical characteristics [43,44]. Orwin and Wadle [44] found that microbial communities in soils with higher N content, but limited C were more resilient to changes caused by drying. The lack of C in soils with high N may result in slow microbial growth or inactivity and this can constitute a survival mechanism to stress conditions [44]. Also, the higher availability of N may have allowed the preponderance of slow-growing microbes that are more resilient to changes in the Piedmont soil. Furthermore, changes in hydrogen ions (H^+^) concentration, which increases with decreasing pH, have also been linked with the presence or absence of soil microbial communities, suggesting that the microbial communities in the two soils may be potentially different [45]. This might be a reason for the observed differences in amendment effect on the viable microbial biomass in the two soils. More so, a global meta-analysis showed that microorganisms are easily affected by biochar in sandy soils than clayey soils [46]. On the whole, it appears that the decrease in the total viable microbial biomass observed in the Coastal soil was largely driven by the changes in bacterial biomass as the fungal biomass remained unchanged (Table 4 and Fig 4). Moreover, the presence of recalcitrant C or toxic aromatic compounds under repeated D-W2 and D-W3 can result in a decline in bacterial biomass [47]. However [48], found no differences in overall microbial abundance following the application of biochar obtained from either corn or hardwood.

### Changes in microbial community composition indices

In our study, shifts in microbial community composition, F:B, Gram-positive (G+):Gram-negative (G-), and saturated fatty acid to unsaturated fatty acid (sat:unsat) ratios were calculated (Table 4). In general, shifts in microbial communities were distinct in the Coastal soil than in the Piedmont soil irrespective of the drying-rewetting cycle indicating the higher sensitivity of microorganisms in the sandy soil as compared to the clayey soil. In the more acidic Coastal soil, the F:B PLFA ratio became broader in biochar amended soils indicating a shift driven by the presence of lignocellulosic compounds that are harder to degrade [49]. Furthermore, our results also showed soil-specific responses to variation in hydrological cycles irrespective of the use of amendments. The F:B PLFA ratio increased in the sandy Coastal soil under either repeated drying and rewetting cycle (D-W2) or constant moisture (D-W3) while in the clay Piedmont soil increase in F:B PLFA ratio was recorded only under D-W3. In soils with higher sand content, the loss of C via heterotrophic respiration is higher compared to clay soils where increased stabilization of C compounds can occur due to protection in soil aggregates or organomineral complex interactions. Averaged over soil types, the lower F:B PLFA ratio in HR treatments suggests a slower decomposition of organic matter under D-W2 than D-W3 (Table 4). It is conceivable that the activities of decomposer microorganisms will be faster as constant moisture persists and labile C compounds in HR residues are used up faster [50]. This can be confirmed by the consistent higher F:B PLFA ratio in both HB- and HA-amended soils under D-W2 and D-W3 and the lack of differences among the treatments in D-W1 (Table 4). A higher F:B ratio suggests efficient C cycling as greater amounts of C are retained in fungal biomass than bacterial biomass and may be beneficial. Overall, our results indicate that both bacterial and fungal communities in the sandy Coastal soil were more sensitive to the use of extraneous organic matter or variability in hydrological cycles as compared to the clay Piedmont soil.

The measured G(+):G(-) PLFA ratios were lower in amended Coastal soil when averaged over hydrological cycles but remained unchanged in the Piedmont soil (Table 4). Likewise, changes in G(+):G(-) PLFA ratios due to moisture-induced stress followed the same trend in both soil types. Like the G(+):G(-) PLFA ratio, the sat:unsat PLFA concentrations were lowest in HR-amended Coastal soils while no changes were observed in the Piedmont soil when averaged over moisture cycles (Table 4). On the contrary, unlike the G(+):G(-) PLFA ratio which remained unchanged irrespective of treatments across the moisture cycles when averaged over soil type, the sat:unsat PLFA concentration was affected during D-W1 and D-W2 but was lowest in HR-amended soils in D-W3. We also found that when averaged over amendment type, the sat:unsat PLFA concentration in both soils was highest at D-W2 but lowest in D-W1 for the Coastal soil and D-W3 for the Piedmont soil.

Both G(+):G(-) and sat:unsat PLFA concentrations have been used to describe microbial shifts in soils in response to nutrient fluctuations or environmental conditions [38,51]. It is known that G(+) bacteria can persist through stressors such as fluctuations in environmental conditions through their ability to form spores and G(-) can flourish under increase labile C and anaerobic conditions [52]. The decrease in the G(+):G(-) ratio in our study hints at increased soil resources in amended Coastal soil and under D-W2 and D-W3. While a previous research showed that repeated dry and wet cycles caused an increase in G(+) bacteria in biochar amended soils [7], changes in the G(+):G(-) bacterial biomass ratio with amendments have been shown to be variable and also associated with resource availability, amendment characteristics, and soil type [38,51,53]. Likewise, the decrease in sat:unsat PLFA concentrations, a stress index, also confirms the increased resources associated with amended Coastal soils, especially under HR [54]. Variation in moisture had a more complex effect on the sat:unsat PLFA concentration. The lower sat:unsat PLFA concentration in the Piedmont soil under D-W3 indicates organic matter as likely used up faster than at D-W2 (Table 4). Taken together, the trend in changes in G(+):G(-) PLFA ratio was more distinct than the sat:unsat PLFA ratio. While the ratios do not indicate the exact changes in microbial community composition, they suggest that the adaptation of soil biota to stress caused by the amendments and moisture cycles were congruent to some extent. The effect of environmental disturbance on soil health is likely to be higher in sandy soils than clay soils due to the higher stability of the active microbial community in clay soils.

### Effects of amendments and moisture cycle on chemical soil health indicators

**Fig 5:**
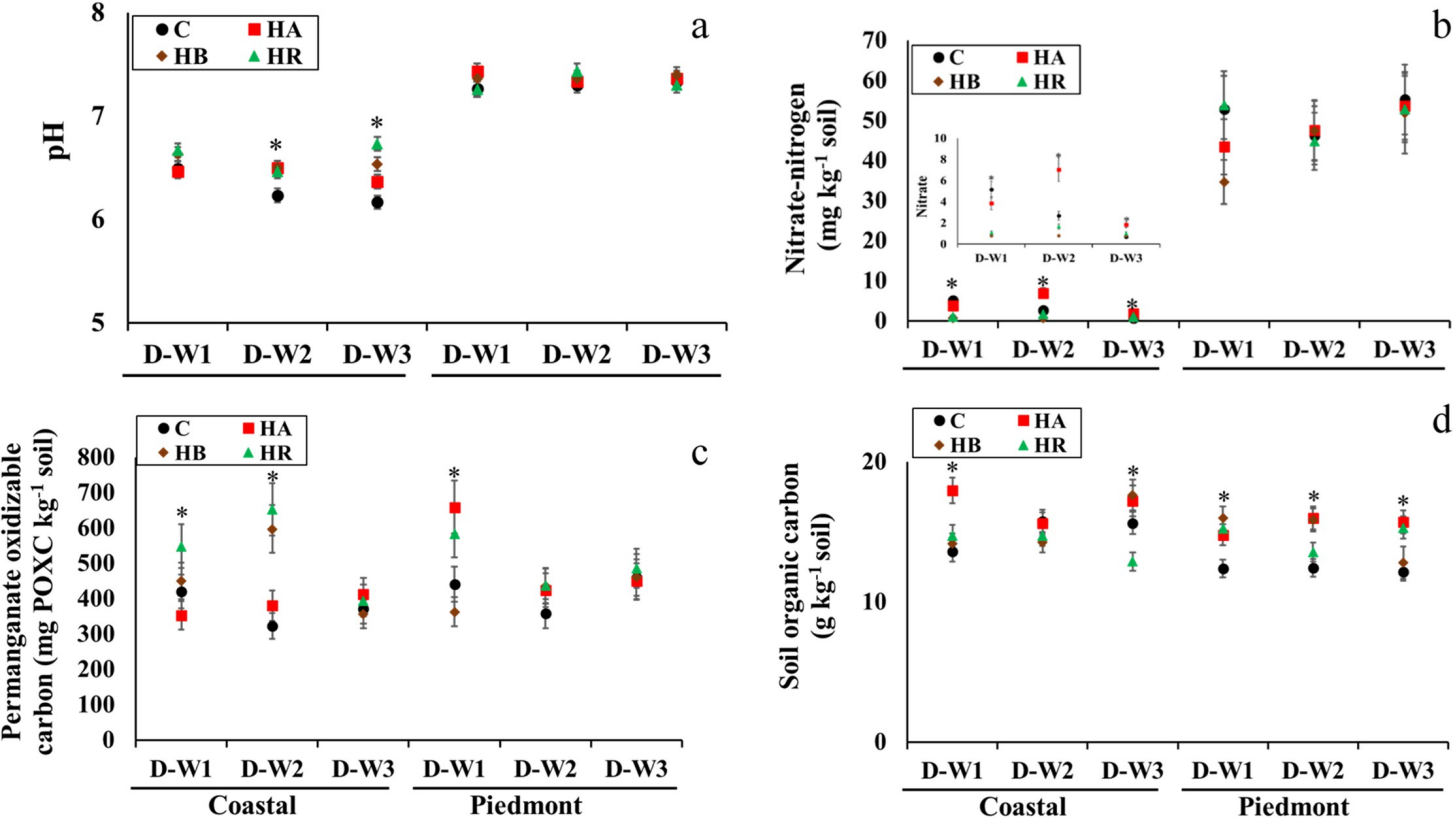
The interaction of soil type × moisture cycle × amendment for the pH, nitrate nitrogen, permanganate oxidizable carbon, and total carbon (panels a, b, c, and d, respectively) in the amended soil mesocosms. Asterisk indicates difference among treatments (α = 0.05), based on three-way ANOVA. Error bars represent standard errors (n = 3). C, control; HR, hemp; HB, hemp biochar; HA, hardwood biochar; D-W1, 30 d 60% WFPS; D-W2, 30 d 60% WFPS followed by 7 d alternate D-W for 42 d; D-W3, 72 d 60% WFPS.

Changes in the soil chemical and biochemical properties are presented in Fig 5. Although there were no changes in pH in the Piedmont soil at the end of the incubation, application of either HR, HB, or HA increased the pH in the Coastal soil (Fig 5A). Contrary to our expectation, biochar had the least effect on pH despite its inherent alkaline pH (Table 2). At D-W2, the amendments had a comparable effect on pH in the Coastal soil, but at D-W3, HR-amended Coastal soils had the highest pH. Biochar is typically known to increase soil pH [50], however, a study found that whether biochar will increase soil pH or not was dependent on the biochar type [30]. The electrical conductivity value of HR was highest among the amendments and could explain the significant effects of HR on pH under constant moisture. The HR residue is expected to decompose faster under constant moisture and given its high electrical conductivity, an indicator of high amounts of soluble salts, it could increase soil alkalinity. The lack of changes in the pH in the Piedmont soil suggests greater buffering capacity which can be linked to its higher clay content or initial pH. Frene et al. [55] reported similar outcomes in sandy soils of North Carolina where the addition of pine-biochar had a slight increase in soil pH.

Our results showed that a higher extractable NO_3_^-^-N was obtainable in HA-amended soils at the end of the incubation at D-W2 and D-W3 in the Coastal soil (Fig 5B). The NO_3_^-^-N in the HA-amended soils (1.8–7.1 mg N kg^-1^) was between 167–175% higher than control soils in the Coastal soil. Contrarily, no changes in NO_3_^-^-N among the amended soils were observed in the Piedmont soil (34–53 mg N kg^-1^). The effect of biochar on NO_3_^-^-N was notable in the Coastal soil with lower inherent NO_3_^-^-N levels (24.3 mg kg^-1^) while there were no distinct differences in the Piedmont soil with higher NO_3_^-^-N values (54.5 mg kg^-1^) (Table 1). Surprisingly, HR and HB amendments had similar effects on NO_3_^-^-N in the Coastal soil which was contrary to the effects of HA. Greater amounts of NO_3_^-^-N were extracted from HA-amended soils compared to HB-amended Coastal soil. Nitrate retention and subsequent availability have been shown to be higher in soils amended with biochar with a low H:C ratio (0.17–0.27) [56]. The HA used in our study has an H:C ratio of 0.17 compared to HB’s and HR’s values of 0.39 and 1.66, respectively. Given that the H:C ratio biochar indicates the degree of aromaticity, biochar with higher aromaticity, therefore, increases NO_3_^-^-N retention and subsequent availability. In addition, the other biochar characteristics such as pore structure and surface area have also been linked to high retention characteristics of biochar [57]. The surface area of the HA used in our study was 656 m² g^-1^ compared to 73 m^2^ g^-1^ for HB, which likely contributed to a higher NO_3_^-^-N concentration at the end of the incubation. Also, the higher amounts of NO_3_^-^-N in HA-amended Coastal soils under continuous drying and rewetting cycle were reduced under continuous moisture (Fig 5B). The reduced NO_3_^-^-N might have resulted from higher microbial N transformation as substrate diffusion is increased at 60% WFPS unlike under D-W cycles. Furthermore, the lack of differences in NO_3_^-^-N values in the Piedmont soil, indicates that soil type had strong effects on NO_3_^-^-N dynamics.

Although the HA biochar used in our study reduced microbial activity and N availability, its high surface area, and low H:C ratio indicates it would be effective for remediating contaminated soils and improving soil health. Several studies [58,59] have linked enhanced soil microbial activity and crop growth to a decrease in the bioavailability of heavy metals in contaminated soils due to sorption on biochar surfaces. Other studies [60,61] that have documented simultaneous decrease in the bioavailability of heavy metals in contaminated soils and enhanced soil microbial activity and N availability had greater (0.6–0.9) biochar H:C ratio as compared to our study, suggesting the likely benefits of HA in contaminated soils.

### Effects of amendments and moisture cycle on biochemical soil health indicators

Coastal soils amended with HR and HB were characterized by high POXC values under D-W1 and D-W2 but not under D-W3 (Fig 5C). On the contrary, Piedmont soil amended with HR and HA were characterized by high POXC values only under D-W1 (Fig 5C). The SOC was significantly affected by the interaction of soil type × amendment × moisture cycle (p = 0.0016) (Fig 5D). In the coastal soil, SOC in HA-amended soil was highest at D-W1, but not statistically different (p>0.05) from control and HB-amended at D-W3. However, in the Piedmont soil, changes in SOC following the application of amendments across the moisture cycles were more dynamic. At D-W1 in the Piedmont soil, all amended soils had similar SOC values which were different from the control. However, at D-W2 in the Piedmont soil, HA- and HB-amended soils had the highest SOC values while at D-W3, the SOC was not different among the amended soils except among HR- and HA-amended soils and control (Fig 5D).

Understanding SOC dynamics under variable moisture conditions in amended soils is important for better management. Drying-rewetting condition (D-W2) seemed to facilitate the loss of active C in clay Piedmont soil compared to sandy Coastal soil (Fig 5C) but under constant moisture (D-W3), the decline in active C followed the same trend in both soils. Although changes in soil moisture were not measured, the high clay content in the Piedmont soils may have contributed to increased substrate diffusion which could lead to increased microbial utilization of labile C and subsequent reduction in active C concentration [62]. This trend is further corroborated by the lack of differences in POXC concentration in both soils at D-W3 where moisture content was maintained at 60% WFPS (Fig 5C). The high POXC values were measured in the Piedmont soil under D-W1 even though enzymatic indicators of labile C for HA-amended soils were low. This suggested that POXC is not necessarily a measure of microbially utilizable C [18]. The divergent effect of moisture events on the active carbon fraction due to differences in soil texture is consistent with the findings of Kirk et al. [16].

While changes in SOC are known to be slow, previous studies indicated that biochar addition mostly increases SOC because of its high OC content per mass [63] even though a decrease in SOC following biochar addition has also been reported [9]. We expected SOC to be highest in HA-amended soils followed by HB- and HR-amended soils given that their C:N ratios are 229, 67, and 46, respectively but there was a lack of clear trend. The use of HA was beneficial for increasing SOC in the sandy Coastal soil under D-W1 while under longer periods of constant moisture (D-W3) the use of crop residues did not significantly increase SOC (Fig 5D). On the other hand, in the clay Piedmont soil, differences in SOC among amended soils were evident at D-W2 and D-W3, with HA having a consistent effect, while HB increased SOC under D-W2 and HR under D-W3. As such, biochar would not always increase SOC as this would depend on the type of biochar as well as the soil type and moisture condition. Furthermore, depending on the physical and chemical properties of the biochar, biochar can differentially shape soil biophysical conditions such as formation and stability of macro- and micro-aggregates, aeration, differences in bonding mechanisms, which could be a possible reason for the differences in SOC [34]. Thus, depending on moisture conditions, determining the type of biochar that would sustain agroecosystem function would depend on the type of soil and biochar [42].

### Relationship between microbial activity and structure, and soil biochemical properties

**Fig 6:**
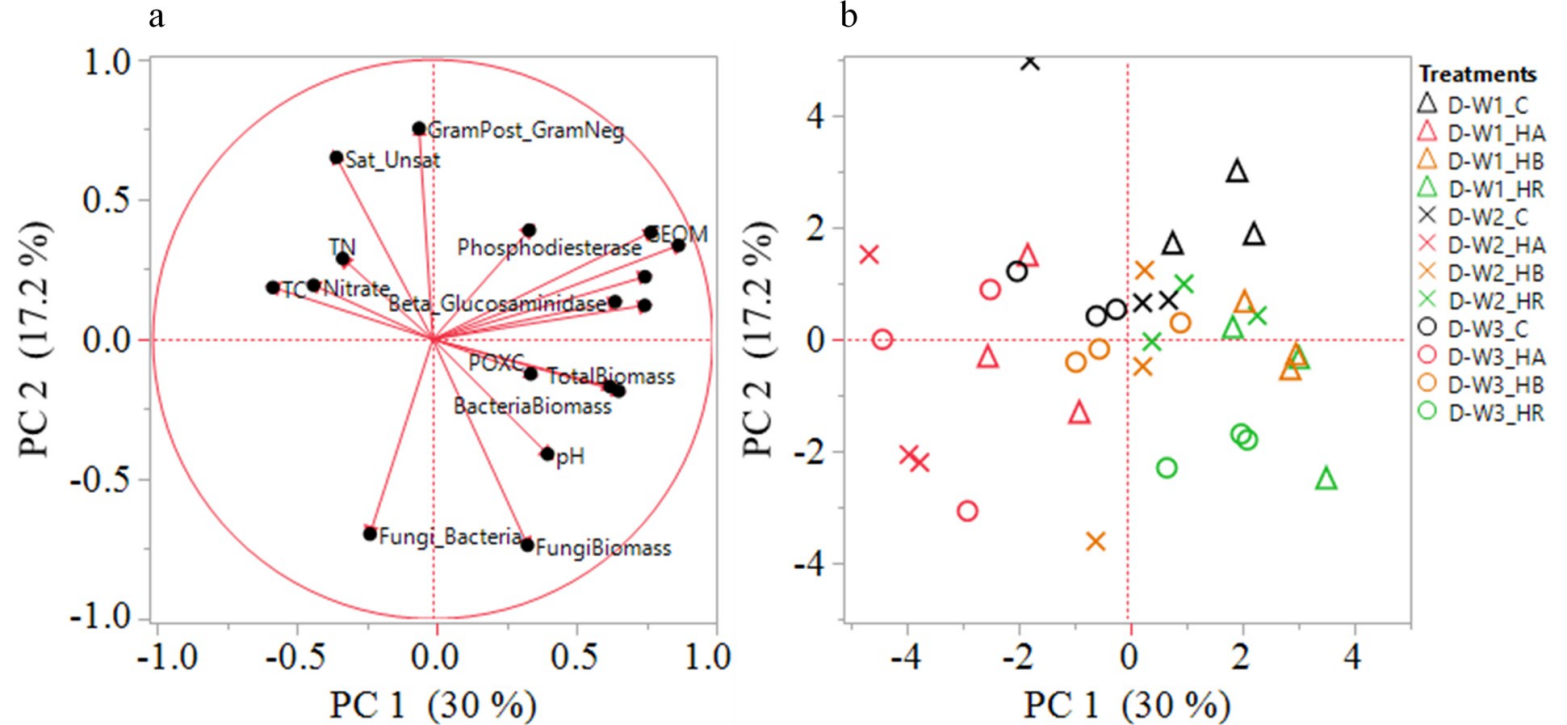
Principal components analysis (panel a: plot of eigen plot of the eigenvectors, panel b: plot of the PC scores) of soil enzymes and phospholipid-derived fatty acid (PLFA) profiles as impacted by amendments and moisture cycles in the Coastal soil. D-W1, 30 d 60% WFPS; D-W2, 30 d 60% WFPS followed by 7 d alternate D-W for 42 d; and D-W3, 72 d 60% WFPS. C, control; HR, hemp residue; HB, hemp biochar; HA, hardwood biochar. GEOM, geometric mean of enzyme activities; POXC, permanganate oxidizable carbon; TC, total soil organic carbon; TN, total nitrogen; GramPost_GramNeg, gram-positive to gram-negative bacteria biomass; Sat_Unsat, ratio of saturated to unsaturated fatty acids.

**Fig 7:**
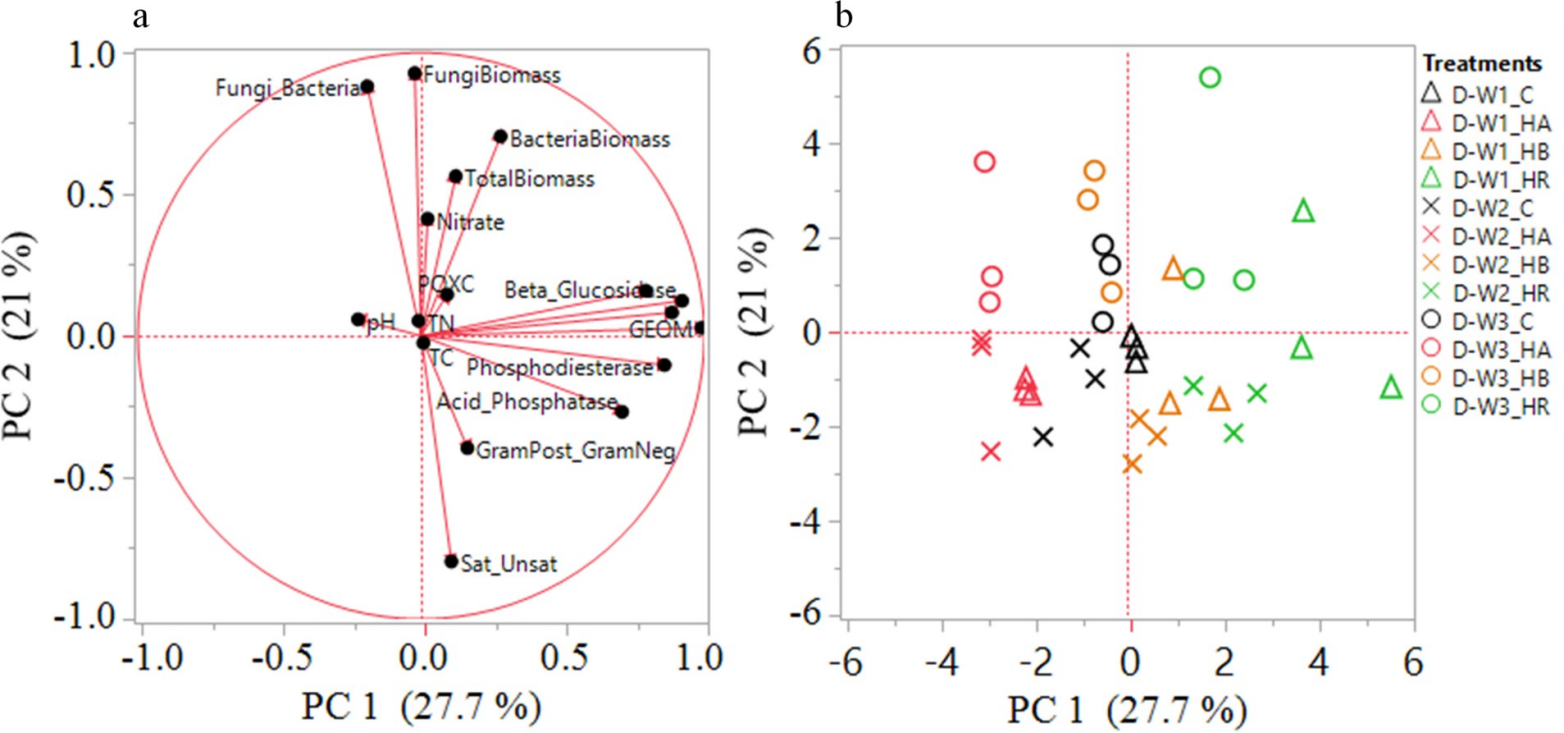
Principal components analysis (panel a: plot of eigen plot of the eigenvectors, panel b: plot of the PC scores) of soil enzymes and phospholipid-derived fatty acid (PLFA) profiles as impacted by amendments and moisture cycles in the Piedmont soil. D-W1, 30 d 60% WFPS; D-W2, 30 d 60% WFPS followed by 7 d alternate D-W for 42 d; and D-W3, 72 d 60% WFPS. C, control; HR, hemp residue; HB, hemp biochar; HA, hardwood biochar. GEOM, geometric mean of enzyme activities; POXC, permanganate oxidizable carbon; TC, total soil organic carbon; TN, total nitrogen; GramPost_GramNeg, gram-positive to gram-negative bacteria biomass; Sat_Unsat, ratio of saturated to unsaturated fatty acids.

**Fig 8:**
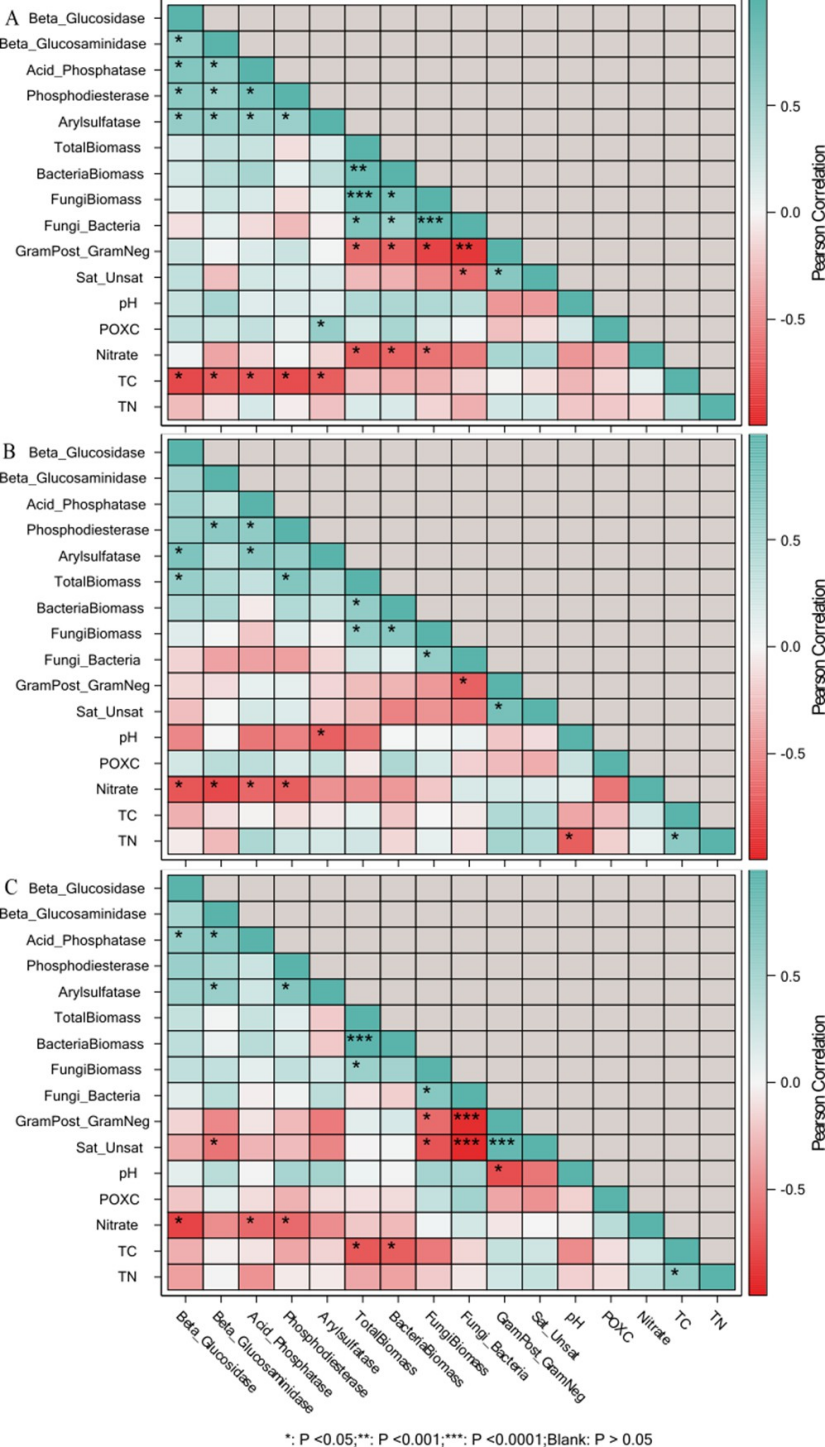
Pearson correlation matrix of soil enzymes, microbial community structure and biochemical properties of soil in Coastal Plain soil in D-W1, 30 d 60% WFPS (panel a); D-W2, 30 d 60% WFPS followed by 7 d alternate D-W for 42 d (panel b); D-W3, 72 d 60% WFPS (panel c). GEOM, geometric mean of enzyme activities; POXC, permanganate oxidizable carbon; TC, total soil organic carbon; TN, total nitrogen; GramPost_GramNeg, gram-positive to gram-negative bacteria biomass; Sat:Unsat, ratio of saturated to unsaturated fatty acids. *p<0.05, **p<0.01, ***p<0.0001.

**Fig 9:**
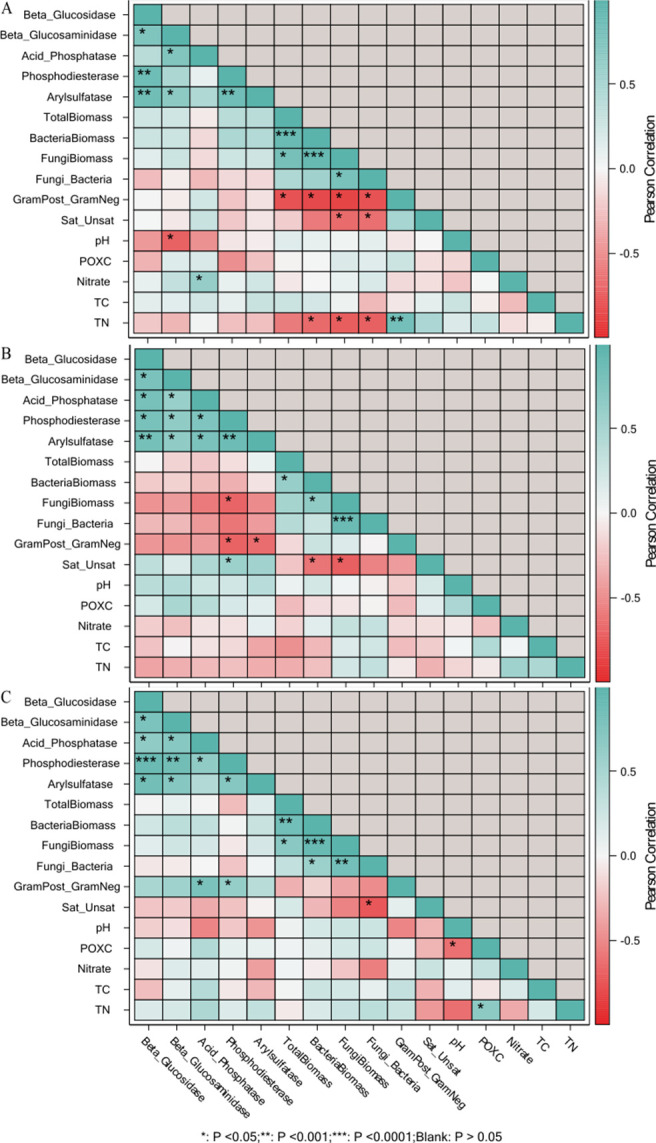
Pearson correlation matrix of soil enzymes, microbial community structure and biochemical properties of soil in Piedmont soil in D-W1, 30 d 60% WFPS (panel a); D-W2, 30 d 60% WFPS followed by 7 d alternate D-W for 42 d (panel b); D-W3, 72 d 60% WFPS (panel c). GEOM, geometric mean of enzyme activities; POXC, permanganate oxidizable carbon; TC, total soil organic carbon; TN, total nitrogen; GramPost_GramNeg, gram-positive to gram-negative bacteria biomass; Sat:Unsat, ratio of saturated to unsaturated fatty acids. *p<0.05, **p<0.01, ***p<0.0001.

The linkages between soil function, community structure, and chemical composition were assessed using PCA and correlation analyses (Figs 6–9). Our indices of soil microbial function were more strongly related to amendment type as compared to moisture cycle. For both soils, enzyme activities were heavily loaded on principal component 1 (PC1) which explained 30% (Coastal) and 28% (Piedmont) of the variance in β-glucosidase (0.336), phosphodiesterase (0.345), arylsulfatase (0.335), the geometric mean of enzyme activities (0.388), total PLFA biomass (0.281), and total PLFA bacterial biomass (0.234) for Coastal soil and β-glucosidase (0.424), β-glucosaminidase (0.366), acid phosphatase (0.327), phosphodiesterase (0.396), arylsulfatase (0.408), and the geometric mean of enzyme activities (0.456) for Piedmont soil. For both soils, soil microbial community structure indices loaded strongly on the principal component 2 (PC2) (Figs 6 and 7 and S1 and S2 Tables). In the Piedmont changes in soil biological and biochemical properties were mainly driven by the HR followed by HB but in the Coastal soil, HB strongly impacted soil biological properties and HA has stronger effects on SOC, total nitrogen, and nitrate concentration (Figs 6 and 7). In addition, while the effect of the moisture cycle was indistinguishable in the Coastal soil, it was fairly distinct in the Piedmont soil (Figs 6B and 7B). Thus, different management strategies would be required for different soils under variable moisture events.

Furthermore, the correlation analysis showed that PLFA biomarkers were weakly associated with measures of nutrient cycling and other studied soil chemical and biochemical variables (Figs 8 and 9). In the Coastal soils, SOC was observed to be negatively correlated with β-glucosidase, β-glucosaminidase, acid phosphatase, phosphodiesterase, and arylsulfatase at D-W1, while at D-W2 and D-W3, nitrate was negatively correlated with the enzymes except for arylsulfatase and β-glucosaminidase at D-W3 (Fig 8). Similarly, G(+): G(-) ratio showed a negative correlation with total PLFA biomass, total PLFA bacterial and fungal biomass, and F:B ratio especially at D-W1 and D-W3. A similar correlation trend was observed for the Piedmont soil as soil enzyme activities were weakly associated with microbial community structure indices. At D-W2, the fungal biomass and G(+): G(-) ratio were negatively correlated with phosphodiesterase and arylsulfatase while sat:unsat ratio and G(+): G(-) ratio at D-W3 were positively correlated with phosphodiesterase activity (Fig 9). This indicated that changes in soil enzyme activities were found to be largely decoupled from shifts in microbial communities [64]. Overall, it appears that changes in bacterial PLFA biomass largely drive the shifts in total microbial PLFA (Figs 8 and 9). Also, consistent with previous studies, changes in soil microbial community structure varied with biochar type and soil characteristics [65,66].

The negative relationship between N and enzyme activity can be explained by the differences in N sorption and retention by biochar which affects microbial activity. This was evidenced by the higher amount of extractable nitrate from HA amended soils. For example, Rajkovich et al. [67] found that biochar addition increased N sorption, which in turn limits microbial activity. In addition, Cayuela et al. [68] also explained that biochar addition might reduce microbial cycling of N. Furthermore, the negative relationship between SOC and enzyme activities reflects the presence of recalcitrant pyrogenic C that could be linked to biochar addition [48]. Our results indicate that both HR and HB would be beneficial to improving soil activity under changing hydrological events.

## Conclusion

Impacts of three different amendment types (hemp residue, hemp biochar, and hardwood biochar) on soil microbial community structure and function, and biochemical soil health indices at three different moisture cycles were examined in a laboratory incubation experiment in two soil types from North Carolina. Our study demonstrates that both hemp residue and hemp biochar occasionally improved soil microbial enzyme activities (*e*.*g*., β-glucosidase, β -glucosaminidase, acid phosphatase, arylsulfatase, and phosphodiesterase), though the extent varied with soil type within the same moisture cycle. On the other hand, the application of hardwood biochar consistently led to a decline in soil nutrient cycling shown by the reduced soil enzymatic activities irrespective of the soil type or moisture cycle because of its high surface area and low H:C ratio. The microbial biomass PLFA revealed unclear effects among amended soils, although changes were more distinct in the sandy soil (Coastal) than the clay soil (Piedmont), and at D-W2 and D-W3, indicating the strong impact of soil type and moisture cycle. It thus appears that under repeated D-W cycle, the application of hemp biochar would likely enhance biological soil health under fluctuating and dynamic hydrological events, especially in coarse texture soils. Based on our current study, the application of hemp biochar has the potential to enhance soil health, considering the positive effect on soil microbial functional diversity and promote soil C accrual in Piedmont and Coastal Plain soils in North Carolina.

## Supporting information

S1 Table Eigenvectors of the leading principal components based on moisture cycle and amendments in the Coastal Plain soil. (DOCX)

S2 Table Eigenvectors of the leading principal components based on moisture cycle and amendments in the Piedmont soil. (DOCX)

S1 File (XLSX)
