# Supplementary material for: Hemp biochar impacts on selected biological soil health indicators across different soil types and moisture cycles
Source: PLoS One. 2022 Feb 28;17(2):e0264620. doi: 10.1371/journal.pone.0264620 (PMC8884510; doi:10.1371/journal.pone.0264620)
Supplement: S1 Table — (DOCX) [file pone.0264620.s001.docx]

**Table S1** Eigenvectors of the leading principal components based on moisture cycle and amendments in the Coastal Plain soil

| Variable | Prin1 | Prin2 |
| --- | --- | --- |
| Beta glucosidase | 0.336 | 0.131 |
| Beta glucosaminidase | 0.288 | 0.079 |
| Acid phosphatase | 0.152 | 0.229 |
| Phosphodiesterase | 0.345 | 0.224 |
| Arylsulfatase | 0.335 | 0.071 |
| Total PLFA biomass | 0.281 | -0.099 |
| PLFA bacteria biomass | 0.294 | -0.108 |
| PLFA fungi biomass | 0.150 | -0.431 |
| F:B^†^ | -0.099 | -0.408 |
| G (+): G(-) | -0.022 | 0.442 |
| Sat:Unsat | -0.153 | 0.381 |
| pH | 0.182 | -0.240 |
| GEOM | 0.389 | 0.197 |
| POXC | 0.155 | -0.072 |
| Nitrate | -0.189 | 0.113 |
| TC | -0.253 | 0.109 |
| TN | -0.143 | 0.169 |

F:B, fungi biomass to bacteria biomass ratio; GEOM, geometric mean of enzyme activities; POXC, permanganate oxidizable carbon; TC, total soil organic carbon; TN, total nitrogen; G (+): G(-), gram-positive to gram-negative bacteria biomass; Sat:Unsat, ratio of saturated to unsaturated fatty acids.
