# Supplementary material for: Hemp biochar impacts on selected biological soil health indicators across different soil types and moisture cycles
Source: PLoS One. 2022 Feb 28;17(2):e0264620. doi: 10.1371/journal.pone.0264620 (PMC8884510; doi:10.1371/journal.pone.0264620)
Supplement: S2 Table — (DOCX) [file pone.0264620.s002.docx]

**Table S2** Eigenvectors of the leading principal components based on moisture cycle and amendments in the Piedmont soil

| Variable | Prin1 | Prin2 |
| --- | --- | --- |
| Beta glucosidase | 0.424 | 0.065 |
| Beta glucosaminidase | 0.366 | 0.084 |
| Acid phosphatase | 0.327 | -0.142 |
| Phosphodiesterase | 0.396 | -0.055 |
| Arylsulfatase | 0.408 | 0.044 |
| Total PLFA Biomass | 0.056 | 0.298 |
| Bacteria PLFA biomass | 0.129 | 0.373 |
| Fungi PLFA biomass | -0.010 | 0.491 |
| F:B^†^ | -0.088 | 0.466 |
| G (+): G(-) | 0.075 | -0.210 |
| Sat:Unsat | 0.049 | -0.421 |
| pH | -0.103 | 0.031 |
| GEOM | 0.456 | 0.015 |
| POXC | 0.043 | 0.077 |
| Nitrate | 0.011 | 0.219 |
| TC | 0.004 | -0.013 |
| TN | -0.004 | 0.028 |

F:B, fungi biomass to bacteria biomass ratio; GEOM, geometric mean of enzyme activities; POXC, permanganate oxidizable carbon; TC, total soil organic carbon; TN, total nitrogen; G (+): G(-), gram-positive to gram-negative bacteria biomass; Sat:Unsat, ratio of saturated to unsaturated fatty acids.
